# Preparing the Implementation of Computerized Adaptive Testing for High-Stakes Examinations

**DOI:** 10.3352/jeehp.2008.5.1

**Published:** 2008-12-22

**Authors:** Sun Huh

**Affiliations:** Department of Parasitology, College of Medicine and Institute of Medical Education, Hallym University, Chuncheon, Korea.

On 13 October 2008, staff members of the National Health Personnel Licensing Examination (NHPLE) of the Republic of Korea visited Hallym University to observe students taking a computerized adaptive testing (CAT) via personal computer. Afterwards, the staff members discussed CAT and its installation with Hallym University faculty and the psychometrician. NHPLE is still undecided about the timetable for introducing CAT to the Korean Medical Licensing Examination (KMLE). Although the KMLE is expected to adopt CAT eventually, implementation after an official announcement could take up to four years. Medical schools can use the period before the announcement to prepare for CAT. To adapt CAT to a high-stakes examination such as the KMLE requires that NHPLE, the presiding organization, conduct studies to construct the best system and bench-mark examples from other countries, such as Canada. The role of the psychometrician is essential to the process. In addition, each medical school should prepare for CAT by first introducing computer-based testing (CBT). CBT has many of the same merits as paper-and-pencil tests (PPT), but may provide a more reality-based clinical situation in the medical institutes. Korea's high-speed internet bandwidths and high-quality hardware provide excellent conditions for CBT, and some schools have already introduced it. After CBT is established, CAT item parameters can be calculated, and the database of items and item parameters can be constructed. One challenge in introducing CAT to individual Korean medical schools is the difficulty of creating stable item parameters, as the number of students in each school is fewer than 150. To overcome this size constraint, medical schools may form consortia to enable the construction of more stable item databases. Open-source CBT and CAT projects already exist at sourceforge. net, which can serve as a good resource for medical schools. Finally, the expertise of psychometricians is essential to the launch of CAT at each school. Many medical schools in Korea have begun to recruit education specialists, but recruiting psychometricians may require more time. These specialists have a theoretical understanding of issues such as item-response theory, models, and other factors that should be considered in applying CAT. If recruitment is not an option, psychometric consultants should be hired. Although implementing CAT at each school may take time, step-by-step preparation is mandatory to facilitate this useful evaluation tool for medical students.

